# “There are still a lot of things that I need”: a qualitative study exploring opportunities to improve the health services of First Nations People with arthritis seen at an on-reserve outreach rheumatology clinic

**DOI:** 10.1186/s12913-020-05909-9

**Published:** 2020-11-25

**Authors:** Adalberto Loyola-Sanchez, Ingris Pelaez-Ballestas, Lynden Crowshoe, Diane Lacaille, Rita Henderson, Ana Rame, Tessa Linkert, Tyler White, Cheryl Barnabe

**Affiliations:** 1 Division of Physical Medicine and Rehabilitation, Faculty of Medicine and Dentistry, University of Alberta, Edmonton, Alberta Canada; 2 Department of Rheumatology, Hospital General de Mexico “Dr. Eduardo Liceaga”, Mexico City, Mexico; 3 Department of Family Medicine, Cumming School of Medicine, University of Calgary, Calgary, Alberta Canada; 4 Division of Rheumatology, Faculty of Medicine, University of British Columbia, Vancouver, British Columbia Canada; 5 Departments of Medicine and Community Health Sciences, Cumming School of Medicine, University of Calgary, Calgary, Alberta Canada; 6 Department of Medicine, Cumming School of Medicine, University of Calgary, Calgary, Alberta Canada; 7 Siksika Health Services, Siksika Nation, Siksika, Alberta Canada

**Keywords:** Indigenous peoples of Canada, Arthritis, Models of care, Qualitative methods

## Abstract

**Background:**

Arthritis is a highly prevalent disease and leading cause of disability in the Indigenous population. A novel model of care consisting of a rheumatology outreach clinic in an on-reserve primary healthcare center has provided service to an Indigenous community in Southern Alberta since 2010. Despite quality assessments suggesting this model of care improves accessibility and is effective in meeting treatment targets, substantial improvements in patient-reported outcomes have not been realized. Therefore, the objective of this study was to explore the experiences of Indigenous persons with arthritis and healthcare providers involved in this model of care to inform the development of health service improvements that enhance patient outcomes.

**Methods:**

This was a narrative-based qualitative study involving a purposeful sample of 32 individuals involved in the Indigenous rheumatology model of care. In-depth interviews were conducted to elicit experiences with the existing model of care and to encourage reflections on opportunities to improve it. A two-stage analysis was conducted. The first stage aimed to produce a narrative synthesis of concepts through a dialogical method comparing people with arthritis and health providers’ narratives. The second stage involved a collective effort to synthesize concepts and propose specific recommendations to improve the quality of the current model of care. Triangulation, through participant checking and discussion among researchers, was used to increase the validity of the final recommendations.

**Results:**

Ten Indigenous people with arthritis lived experience, 14 health providers and 8 administrative staff were interviewed. One main overarching theme was identified, which reflected the need to provide services that improve people’s physical and mental functioning. Further, the following specific recommendations were identified: 1) enhancing patient-provider communication, 2) improving the continuity of the healthcare service, 3) increasing community awareness about the presence and negative impact of arthritis, and 4) increasing peer connections and support among people living with arthritis.

**Conclusions:**

Improving the quality of the current Indigenous rheumatology model of care requires implementing strategies that improve functioning, patient-provider communication, continuity of care, community awareness and peer support. A community-based provider who supports people while navigating health services could facilitate the implementation of these strategies.

**Supplementary Information:**

The online version contains supplementary material available at 10.1186/s12913-020-05909-9.

## Background

Arthritis is an umbrella term used to identify a collection of over 100 musculoskeletal conditions, including highly prevalent conditions such as osteoarthritis and rheumatoid arthritis [1]. Arthritis is among the major causes of disability globally [2, 3], and its disabling effects are heightened in populations that experience higher burden of disease related to health inequities, such as Indigenous communities. Several recent studies have shown that rheumatic diseases are highly prevalent in Indigenous peoples of Latin America, Australia, Canada, New Zealand and the United States, causing severe pain, joint stiffness, disability and high utilization rates of acute healthcare services [4–6].

Specifically in Canada, Indigenous peoples have a higher prevalence and severity of arthritis than the non-Indigenous population [7]. Inflammatory arthritis and osteoarthritis are the most common chronic diseases affecting Indigenous peoples [8] and their disabling effects are distinctly higher than in the rest of the population [9]. In the province of Alberta, these health inequities are in part related to difficulties in accessing sufficient healthcare [10], specialists [11] and medications [12]. A rheumatology outreach clinic, embedded in a primary healthcare center, was established in 2010 in the Indigenous rural reserve of Siksika Nation, Alberta, as an Indigenous rheumatology model of care to mitigate some of these inequities.

Siksika Nation, a member of the Blackfoot Confederacy, is located in Southern Alberta and has approximately 6000 members [13]. A seven-year quantitative longitudinal study of patients with inflammatory arthritis from the Indigenous rheumatology model of care in Siksika demonstrated success in meeting the Canadian Rheumatology Association’s performance indicators related to patient reassessment and disease-modifying anti-rheumatic drug initiation [14], along with significant improvements in objective measures of disease activity [15]. Nevertheless, signals for further improvement in enhancing access to the service were seen, and no significant longitudinal changes in patient-reported outcomes of pain and function were observed [15]. Consequently, the health service team proposed to investigate additional health service intervention models of care to achieve improved patient outcomes.

The main objective of this study was to understand what is required to improve the current Indigenous rheumatology model of care in Siksika Nation. This objective was achieved through the specific objectives of a) exploring experiences from Indigenous people affected by arthritis who had used the model of care and their perspectives on how this should be improved; and b) exploring service providers’ experiences and opinions on how to improve the care of people living with arthritis in this Indigenous reserve.

## Methods

### Design

This was a narrative-based qualitative study conducted under the tenets of social constructivism [16]. Narratives reflect socially constrained ways of acting and making sense of the world [17]. Theoretically, this study is grounded in the assumptions of narrative qualitative inquiry, including the principle that speakers “construct” events through narratives rather than simply refer to them [18]. We utilized a hermeneutical approach on a dialogue between extended narratives about experiences of care and narratives about service provision, as a way to produce interpretations grounded in human actions and cultural differences. Consequently, our interpretations are social co-constructions that help understand people’s experiences of receiving and delivering care within a specific cultural context, allowing the identification of actions for change.

### Setting and population

Siksika Nation is a Blackfoot rural reserve, accommodating over 3000 people on an area of 702 km^2^ [13, 19], and has a dedicated primary health service, Siksika Health Services. Siksika Health Services offers self-determined comprehensive professional, clinical, community health and wellness, homecare and assisted living services through agreements with the federal (Department of Indigenous Services Canada) and provincial (Alberta Health Services) health service bodies. This center is an example of healthcare innovation that has improved access to appropriate and holistic healthcare for Siksika members through established collaborations with regional, national and international organizations [20].

A specialist-led rheumatology outreach clinic was established in the Siksika Health and Wellness Center in 2010 as an Indigenous rheumatology model of care. From October 2010 to February 2018, nearly 300 new patient consultations were completed in this clinic, with nearly 1000 visits provided. The most common arthritis types seen in this clinic are osteoarthritis, inflammatory arthritis, rheumatic regional pain syndromes (i.e. bursitis, tendinitis, rotator cuff syndrome, plantar fasciitis and nerve entrapments), patellofemoral syndrome and mechanical low back pain. One third of the patients in this clinic have other chronic comorbidities, such as diabetes or hypertension and on average they have a moderate to severe disability as measured by the Health Assessment Questionnaire- Disability Index.

A purposeful sampling strategy was implemented to include a wide variety of perspectives on living and caring for arthritis in the community. This strategy selected people with arthritis attending to the Siksika rheumatology clinic, people providing services within Siksika primary health center and people involved in services provision to Indigenous people at the provincial (i.e. Alberta Health Services) and national (i.e. Health Canada) levels. A snowball sampling technique [21] was utilized through asking ongoing participants to recommend people that could meaningfully add to the information we were collecting. Sampling was conducted until reaching thematic saturation [22] or no new general narratives about receiving or providing healthcare were obtained. No exclusion criteria were considered.

### Ethical considerations

This study was approved by Siksika Health Services leadership, through a Memorandum of Understanding, and by The University of Calgary Conjoint Health Research Ethics Board (REB 15–1961). All participants provided consent after the information about the study was carefully explained.

### Data collection methods

One researcher (ALS) conducted individual in-depth interviews at participants with arthritis’ homes or at health professionals/administrators’ workplaces from October 2015 to March 2016. Extensive field notes were taken during and after each interview describing the circumstances in which the interviews were conducted and immediate impressions about the interactions between interviewer and participants.

Two interview guides were utilized to facilitate storytelling, one for people with arthritis (Additional file 1) and one for health providers (Additional file 2). The structure of these interview guides was informed by a pre-defined idea that case management models, or collaborative processes of assessment, planning, facilitation, care coordination, evaluation and advocacy for health services, were ideal to address arthritis patients’ needs. This idea emerged from a literature review that demonstrated a positive impact of case management models on health outcomes through fostering knowledge exchange, decision-making support, mediation with health providers, and system navigation facilitation [23, 24]. Health providers were interviewed before people with arthritis as we required time to build trust with the community and to get the support from health providers to identify and invite people with arthritis to participate in this study.

In the case of health providers, the interviewer asked them to share stories about their practice within the area of Indigenous health. They were asked to reflect on existing health inequities faced by Indigenous Peoples with arthritis and other chronic diseases along with the idea of utilizing a “community case management” model of care to address these inequities. Finally, they were asked to give an opinion about actions for change that they would expect to see within a community intervention.

In the case of people with arthritis lived experience, the interviewer invited them to share stories about their illness trajectories, including obtaining medical services, illness impact and experiences communicating with rheumatologists. Then, they were asked to reflect on these trajectories and to comment on their current strengths and needs. Similar to the interviews with health providers, they were asked on their opinions about the idea of implementing a “community case management” model to address their needs. Finally, they were asked to provide opinions on expected actions for change along with an invitation to participate in a decision-making process to implement improvements to their care.

The interview guides were continuously modified as new narratives were emerging, in order to improve the questions that provoked relevant stories given participants’ roles and positions regarding arthritis care. Interview guide modifications allowed for flexibility to recognize and accommodate for unexpected stories that were linked to the original questions.

### Researchers’ characteristics

The researcher who conducted all interviews and organized the data (ALS) is a physiatrist with a PhD in rehabilitation sciences with training in qualitative methodology. This qualitative study was part of his postdoctoral fellowship and he was not directly involved in the clinical care of people living with arthritis in Siksika. Our research team is composed of individuals from different disciplines (i.e. rheumatology: IPB, DL, CB; family medicine: LC; medical anthropology: RH; healthcare administration: TW) and both Indigenous (TW, LC, CB) and settler (DL, RH, ALS, IPB) backgrounds, all of them committed to reduce the health inequities faced by Indigenous Peoples. The inter-disciplinary team approach taken in this study, allowed for a deeper results interpretation and formulation of stronger recommendations for change in the existing model of care. The research team entered the process with the belief that a case management model applied at the community level could help improve the quality of care of people living with chronic diseases. The latter belief strongly influenced on the implementation of the interviews and on the analysis. Consequently, our results and interpretations come from an authoritative voice [18], which separates the voice of participants from the voice of the researchers, focusing on what was relevant for the research objectives, simultaneously considering and respecting participants’ voices.

### Data processing

All interviews were audio-recorded and transcribed verbatim by a transcriptionist. Field notes were then incorporated into the transcripts to provide context for each interview, facilitating the codification process. Transcripts were anonymized and transferred to specialized software for qualitative data organization and management (HyperResearch version 3.7.3).

### Data analysis

The analysis was conducted in two main stages. Stage one was performed by one researcher (ALS), using Chase’s narrative strategy [18]. This strategy consisted of listening to each interview, identifying plots or stories that allowed for the movement of thought regarding health care improvement. Health providers and patients’ narratives were separately analyzed. Once the plots were identified and organized, a constant dialogic comparison analysis was conducted to identify similarities and differences between patients and health providers’ narratives, which allowed the construction of sensitizing concepts [25], or ideas that could help improve the model of care. Finally, definite concepts [25], or ideas grounded in meaningful stories, were constructed with the help of an adaptation of the “USAID Logical Framework”, which is widely used by the international social development community to help design projects and achieve measurable results (Additional file 3) [26]. Consequently, the result of this main stage was a narrative synthesis organized under the sequence of needs, purpose, functions and roles to address the needs.

The second analytic stage was conducted by all the authors of this manuscript and implied a collective effort to decolonize the findings and interpretations constructed during the previous stage, identifying themes related with health services improvement. Engaging Indigenous and non-Indigenous scholars in a collective analysis to question the fit between the data and the results/interpretations produced during stage one allowed for the identification of over-interpretations and misinterpretations. The formation of a cross-cultural partnership among these scholars achieved one of the goals of decolonization [27]. Even though the objectives of this study are highly pragmatic (i.e. to identify actions for health service improvement), we could not obviate that this study was situated in an Indigenous context where the historical forces of oppression and colonization still exist. Consequently, all researchers participating in the collective analysis tried to include a social justice perspective to fulfill one of the main principles of decolonizing research [28], favoring equity for Indigenous populations when interpreting results and proposing actions.

### Techniques to enhance trustworthiness

The trustworthiness and credibility of our results was enhanced through a member checking process, the completion of an audit trail and an investigator triangulation strategy [29]. The member checking process involved an in-person group meeting discussion to confirm the results and interpretations generated during the first analytic stage with some of the study participants (18 in total, ten patients and eight health care providers/administrators). All our analytic decisions can be traced back through an audit trail, composed by a series of word, excel and power point documents. Finally, the second stage of the analytic strategy involved perspectives from different researchers, Indigenous and non-Indigenous, in an effort to ensure the convergence of the final interpretations.

## Results

Thirty-two in-depth interviews were conducted with 32 individuals, ten with arthritis lived experience and 22 health providers/administrators. Six participants had rheumatoid arthritis, one participant psoriatic arthritis and two participants osteoarthritis. Their experience living with these conditions ranged between two to 25 years. Fourteen of the health providers were directly delivering services to people with arthritis and other chronic diseases in Siksika (i.e. one rheumatologist, two family physicians, four nurses, four wellness-counselors, one community health worker, one occupational therapist, and one pharmacist). In addition, five health providers were performing mainly administrative roles within Siksika Health Services, two health providers were working in Indigenous health departments within regional and federal health institutions (i.e. Alberta Health Services and Health Canada) and one health provider was working as an Indigenous Health academic at the University of Calgary. Seven health providers were Indigenous. Interviews to health providers were completed first, followed by interviews with people with arthritis, thematic saturation was achieved around the interview of the second last person with arthritis (i.e. interview number 31).

Overall, participants suggested improving the model of care for people living with arthritis in Siksika required an increase in patient-centeredness, entailing a need to focus on persons’ individual and collective functioning, or on supporting individuals and group of individuals to increase their meaningful participation in life. This could be achieved through 1) enhancing patient-provider communication, 2) improving continuity of healthcare services, 3) increasing community awareness about the presence and negative impact of arthritis, and 4) increasing peer connections and support among people living with arthritis in this community. The need for case management and patient navigation was supported in the narratives and participants suggested the implementation of these elements in the rheumatology model of care through the figure of a community facilitator. Following is a description of the theme on functioning (i.e. what is needed) along with a description of the themes on how to improve the model of care at this Indigenous rheumatology clinic along with their respective representative quotations.

### Overarching need: to improve the model’s person-centered approach considering the importance of function

*“I just need … getting a job and become a functional part of society … just to be useful again …*” *(Man living with RA for 3 years).*

The majority of narratives from people with arthritis regarding their main needs involved stories of losing function or disability and journeys directed to overcome this. Some of these narratives focused on the need to improve their individual physical capacities to do what they want to do.

“*… physiotherapy … something … to move [my] joints … I want … to see a program incorporating exercises … to keep … mobile … and not in so much pain …” (Woman living with RA for 5 years).*

In addition, some narratives focused on the need for environmental modifications, so it is easier to participate in meaningful activities within the community.

*“I’m scared to fall when I go out because the ground is so uneven... so I’m limited to what I can do” (Woman living with RA for 20 years).*

Only two of the health providers/administrators mentioned the need to focus on people’s function and only focused on the need for physical therapy, without considering the complexity behind community participation.

*“… physiotherapy. … that’s something we really need here … So everything is in one spot.” (Nurse working at the arthritis clinic).*

The following themes represented main strategies to address the overarching need for improving the current rheumatology model of care:

1. *Enhancing communication between patients and health providers*

Focusing on the dialectic relationship between patients’ and providers/administrators’ narratives uncovered a theme related to the importance of improving understanding between them. Specifically, the narratives suggested two main strategies to foster mutual understanding:

- increasing patients’ health literacy *“Advocacy and interpretation bilaterally of not just health literacy but also patient context, back and forth between service providers and the patient” (Family Physician)* and
- increasing health providers’ knowledge about Siksika culture. *“Yeah … we should educate health providers about the Siksika culture.” (Woman with RA for 22 years)*

People living with arthritis identified the need to learn more about their illness.

“*We need to … be educated on our illnesses … what kind of job we need … more information … help me learn and be better” (Man with RA for 3 years).*

This need to increase their health literacy was not exclusive of biomedical knowledge but included a strong desire of integrating Siksika knowledge on healing.

*“That’s part of the healing journey...It’s something good to look back on the Medicine Wheel (the medicine wheel is a modern Indigenous teaching tool utilized to understand and enact the process of healing and wellbeing) …” (Woman with RA for 1 year).*

These narratives had a strong relationship with personal identities:

*“Once you find out who you are, you feel proud and could connect with your spirit and heal” (Indigenous mental health counsellor).*

In addition, some patients’ narratives strongly aligned with the idea of educating non-Indigenous health providers on Siksika’s culture, specifically on the cultural aspects related to health and healing.

*“There is a culture sensitivity issue … [for example] I wanted to see a female [health provider], us native women want to see female doctors [specially] when is something private …” (Woman with RA for 22 years).*

Overall, providers/administrators’ narratives agreed with the need to increase patients’ health literacy in order to improve communication.

*“Need communication. That they let us know what we need … whether family and the patient are educated on their illness, they can make better decisions for themselves and take ownership …” (Family physician).*

In addition, there was agreement in providers/administrators’ narratives about their need to better understand Siksika culture in order to promote a more open communication.

*“[we need] … help with that honest conversation of what’s been going on. Because of course we don’t know what we are not told …” (Clinic administrative team leader).*

*“… If people [health providers] can’t understand and have not experienced the spiritual aspects of healing, then it will be very difficult for them to understand the indigenous perspective and beliefs.” (Indigenous mental health counsellor).*

2. *Improving the continuity of health care services*

Several narratives from people living with arthritis focused on experiences of being left behind with a diagnosis of arthritis, which is difficult to understand, producing confusion on how to live with this condition.

*“… Oh you have arthritis; the doctor will tell you that. And you just don’t really understand what it is … there was a three-month time period from seeing the rheumatologist. And in that time period I had major joint swelling … in the hands. Basically, I had really nobody to see. Like to consult with and I had to wait for my next appointment.” (Man with psoriatic arthritis for less than 1 year), “… I just felt like I got dropped … family doctors … do not have as much knowledge about a certain disease … he did not know what medications [I needed]” (Woman with RA for 5 years).*

These narratives contained a plot of “interrupted care”.

“*Because the rheumatologist comes out once a month. And in between that time there’s patients that need that care. Like you know say for instance, when I was first diagnosed with arthritis there was a three-month time period from seeing the rheumatologist. And in that time period I had major joint swelling, like in the hands. Basically, I had really no body to see. Like to consult with and I had to wait for my next appointment” (Man with psoriatic arthritis for less than 1 year).*

This clearly implies a need to improve the continuity of management between primary and specialized health care services. In addition, some narratives suggested the need for patients to better understand their condition and to know how to live with it as best as possible through continuous support.

*“I get to see my specialist once in a great while … and people live with arthritis 24 seven …” (Woman with RA for 5 years).*

*“we really need someone to be there … look at the appointments … if they have questions they can ask that … but has never happened …” (Woman with RA for 20 years).*

There was overall agreement among people with arthritis’ narratives that improving their continuity of care is not just a matter of increasing access to services but is essential to be able to trust on the non-Indigenous people who provide these services.

*“You have to be able to trust. You know, with a non-Native and a Native. You have to build that trust. Because a lot of Natives don’t trust white people or any other nationality. Because of what happened years ago, the residential schools and all that …” (Woman with RA for 20 years).*

Providers/administrators’ narratives agreed on the importance of increasing continuity of care through closer monitoring of disease management and outcomes as well as to improve the communication and bilateral understanding of arthritis in order to achieve a more patient-centered practice and a successful care plan implementation.

*“… trying to check in with the patient [after appointments] on a repeated basis to just to see … did any of your medications for your other conditions change? what was suggested for your care plan from your family physician?” (Community health worker).*

*“..that the … utilization of health services by people while navigating the system with different professionals do not fall through cracks …” (Community health administrative leader).*

A fundamental aspect to improve the benefits of this monitoring expressed in providers/administrators’ narratives was coordination of different services through an efficient flow and integration of information.

*“… one major role of the case manager model should be having one point of entry for all the services improving the coordination of services …” (Rheumatologist).*

*“I think that if there was a model that made everything a little bit more cohesive you know, between specialists, patients, family physician and other healthcare professionals.” (Family physician).*

*“Well I think there absolutely needs to be … awareness of what each person is doing, right?” (Nurse).*

3. *Increasing community awareness*

There was a general consensus among participants with arthritis on the importance to increase community awareness about the existence and impact of this chronic disabling condition. Practically, all arthritis participants’ voices clearly expressed a need to be understood by the rest of their community members. Specifically, their narratives implied a perception that the community does not validate their health problems.

*“[having] arthritis … groups … would be easier to associate with other people with arthritis cause they know exactly what are you going through [and help] becoming more acceptable in the society to have arthritis … (Woman with OA for 10 years)”.*

That produces an experience of not being supported.

*“… people do not understand that arthritis just does not go away … [we need] support outside the family, to other people to understand what I go through every day … to be in pain most of the time … [I need] someone to talk to besides the family …” (Woman with RA for 31 years)*.

In addition, some narratives expressed frustration for not being fully understood by the community.

*“I am sick of people … [answering to my disclosure] I have rheumatoid arthritis … oh yeah I think I have it in my wrist … YOU DON’T have arthritis, honey … public awareness …” (Woman with RA for 5 years).*

The need to increase community awareness about the existence and impact of arthritis in the community was not expressed in the narratives of providers/administrators.

4. *Increasing peer connections and support*

Most participants with arthritis identified the need to know other people living with the illness as a way to feel understood and to learn how to live better with this condition.

*“… one of the most important things is to have a support network with like people …” (Woman with RA for 5 years).*

*“… to talk to somebody I don’t know but will understand … who knows what I’m talking about … what I’m going through … we could exchange what’s helpful, what to do. …” (Woman with RA for less than 1 year).*

*“… doctors just read symptoms in a book they don’t actually live it … talking to someone with the illness can help me learn and be better …” (Man with RA for 2 years).*

Moreover, the need for building a community of peers living with arthritis was seen by some participants as a way to increase their collective strength, which will result in greater benefits for individuals than just facing this illness alone:

*But there is still a lot of things that I need. I would like to have a support group where we sit and talk about our difficulties … Just getting the people with arthritis more involved … And we need more interaction with everybody that has arthritis, not just one with the doctor. We need more people. (Woman with RA for 31 years).*

Some of the providers/administrators narratives also supported the need to increase community peer connections and support for people living with chronic diseases:

*“We were hoping to start up even just a walk-in support group … But not just the nurse practitioner or the family physicians that are there.. But... support group ...that works with (people with) chronic diseases.” (Community nurse).*

However, the overall narratives from providers/administrators did not include this need as a priority to improve the lives of people living with arthritis in the community.

5. *Need for a community facilitator*

Narratives from both participant groups expressed agreement with the idea of having a community member facilitating actions to address the needs, especially the ones related to improving patient-provider communication and continuity of care. Specifically, for participants living with arthritis the most important aspects of having a community facilitator were the opportunities to improve illness understanding and obtain support to effectively implement care plans.

*“I would like to have another native helping me understand my disease and my treatments …” (Woman with OA for 7 years).*

*“Support to … the people … … like the daily basis … the doctor’s appointments … getting … what you need to get better.” (Woman with RA for 20 years).*

Whereas, for providers/administrators, the essential roles of a community facilitator were to facilitate the communication between patients and providers, increase monitoring of health issues and support system navigation to address them.

*“(Someone to) … bridge the gap between the community and the specialist.” (Family physician).*

*“… trying to check in with the patient … on a repeated basis to just to see … did any … medications … other conditions change... what was suggested for your care plan …” (Rheumatologist).*

*“… So to inform the client as to what they need, who they need, and how they are going to get those services.” (Occupational therapist).*

## Discussion

The main findings of this qualitative study provide understanding on five principal needs to improve the healthcare of people living with arthritis in Siksika Nation. The importance of patient-centeredness in the context of functioning, patient-provider communication, continuity of healthcare services, community awareness on the presence and negative impact of arthritis, and peer support was underlined. All themes identified in this study contained distinct perspectives that clearly differentiated the overall narrative of people living with arthritis from the overall narrative of health providers and administrators. This demonstrates the importance of performing dialogic interpretations that will result on deeper understanding of a healthcare model’s operations, allowing for patients’ voices to guide and shape the necessary actions to improve it.

### Improving function

Improving function, either through enhancing body capacity to perform meaningful activities or through modifying the environment, was a main need perceived by the participants with arthritis in this study. This result aligns with what has been consistently reported in the worldwide literature, which shows that in comparison to the general population, people living with arthritis consistently experience higher rates of disability [30, 31]. The effect of the environment on disability has been reported in other qualitative studies conducted with Indigenous people living with arthritis [32].

Focusing on disabling environments force to pay attention to structural determinants of disability, demanding a deeper reflection on the health inequities faced by Indigenous Peoples in Canada. Structural factors such as employment, education and community life disruptions have been associated with the presence and impact of disability [31, 33] and we feel these are important environmental factors to consider the arthritis related disability in Siksika. Moreover, utilizing a bio-psycho-social framework of functioning, such as the one proposed by the World Health Organization (WHO) (i.e. International Classification of Functioning, Disability and Health [ICF] [34]) can help to better understand the disabling effects or arthritis in this Indigenous community, allowing to innovate and implement appropriate interventions.

### Improving communication

Improving communication between patients and their health providers was a fundamental need identified in this study and was mediated by a need to improve mutual understanding. Establishing trust-based relationships between providers and patients with arthritis have been found important in decision-making process regarding pharmacological management [35]. Our findings support this notion, suggesting that trust is an essential component to improve mutual understanding between patients and providers. Mutual understanding implies a bilateral openness to listen and learn from each other, implying a need for providers to modify their usual paternalistic approach to care in which unilateral decisions are made for patients and to engage in an open and honest conversation about illness beliefs and the connection of these beliefs with management decisions. Indigenous-led strategies to promote mutual understanding in cross-cultural settings, such as the “clinical yarning” person-centered framework, have been proposed to establish trust-based relationships between patients and providers [36].

Some qualitative evidence suggests that “being understood as a person and not merely as a disease” is an important aspect of the patient-provider relationship for individuals living with arthritis [37]. This aspect is even more relevant in the context of transcultural encounters, as has been suggested that the basis to a fruitful relationship between providers and patients from different cultural backgrounds relies on establishing a communication pathway that includes self-awareness, respect, adaptability, active listening, recognition of differences and negotiation-collaboration strategies [38].

Moreover, our results showed that improving communication between patients and providers require also an improvement of patients’ health literacy and this not only encompassed the need for bio-medical education on disease but a more holistic perspective that integrates Indigenous knowledge on healing. The Truth and Reconciliation Commission of Canada (TRCC) has echoed the importance of incorporating Indigenous knowledge into the current health practices for Indigenous Peoples [39]. In fact, recommendation number 22 of the TRCC report specifically calls for those involved in the Canadian Health system to integrate Indigenous knowledge in the health management for those individuals who request it, always in collaboration with Indigenous healers and elders [39]. In addition, integrating local knowledge to mainstream health care services has been shown effective to manage some acute [40] and chronic [41] conditions worldwide. In addition, the importance and cost efficacy of a “holistic” approach to manage chronic conditions, such as chronic pain has been well established [42].

### Improving continuity of care

Improving continuity of care was an essential need found in both patients’ and health providers/administrators’ narratives in this study. The positive impact of the “continuity of care” concept in health, defined as a “coherent care of a person over time and setting” on the chronic disease management has been widely commented in the literature [43]. Nevertheless, understanding what type of continuity of care (i.e. relational vs. informational) is associated with determined health outcomes has been difficult. Our findings reflected this situation as on one hand, the narratives from people with arthritis demonstrated the importance of relational continuity of care through a clear desire of going beyond access to services to the establishment of trust-based relationships with health providers; and on the other hand, providers/administrators’ narratives focused on the informational aspect of continuity of care as they put higher emphasis on disease monitoring embedded in an efficient communication network that involves all health providers participating in the care of a determined individual.

A meta synthesis of narratives from people living with multiple chronic health problems showed that having a single trusted clinician that partners for decision making and system navigation is at the core of what people perceives as an adequate continuity of care [44]. Considering this evidence and the results of our study, it seems that establishing coherent and long-term relationships of trust with providers is more important for people with arthritis than having a good flow of information for disease monitoring purposes, as was mainly proposed by providers and administrators.

In the context of Indigenous Health, the importance of relationships is salient, as one qualitative study with Indigenous children in Australia clearly exemplify where strong relationships between patients and providers resulted as the essential ingredient for therapeutic success in occupational therapy [45]. Moreover, establishing effective and consistent processes to monitor and communicate illness progression and complications among providers is important, especially for the implementation of health-related solutions in a timely fashion. Finally, the concept of continuity of care could be revised and incorporate more emphasis on the importance of relationships, especially when focusing in interculturality contexts.

### Increasing community awareness

People living with arthritis strongly identified the need to increase community awareness about the existence and impact of this chronic and disabling condition in their community. Participants living with arthritis clearly voiced that they don’t feel understood and supported by their community, which creates negative feelings of abandonment and shame. Some of the narratives analyzed included tales of people who refused to come out and disclose having arthritis due to fear of being labeled as a weak person.

These narratives underline the presence of an “anticipated stigma”, which consists of people with arthritis feeling other people in their community see them as disease-simulators who are looking for justifications for not engaging in productive activities or to justify their “laziness”. One qualitative study conducted in this same community showed that people with arthritis commonly “tough it out” and have a hard time accepting they have a health problem [10], which could be further explained by our findings on the perception that community do not understand their experiences of pain, disability and disease. In addition, a recent qualitative study conducted in a similar population with arthritis revealed that drug-dependency stigma negatively effects on pharmacological treatment preferences [46].

The concept of anticipated stigma has been described in the literature of chronic disease and has been identified as one of the central components of illness-generated stigmatized identities with strong associations with psychological distress [47]. The consequences of the anticipated stigma are poorer health outcomes as well as delayed and interrupted healthcare utilization [47], which in our case contributes to understanding the low efficacy to improve health perception and quality of life in patients involved in the arthritis model of care at Siksika Health Center.

The fact that none of the providers/administrators who participated in this study identified the need to increase community awareness on arthritis as a priority, suggests a lack of understanding of patients’ experiences in this community. This situation has also been documented in the literature as even though community awareness has been identified as a key factor to decrease the disabling effects of arthritis by health providers and administrators, most strategies to address the problem of arthritis have been typically focused on preventing and or managing the disease without considering the erroneous perceptions about this condition in the community at large [48]. The consequence of this low emphasis on community awareness and health promotion of arthritis is low engagement in advocacy efforts to help create societal changes towards building communities that are more supportive and respectful of people living with arthritis and their limitations. These advocacy efforts should include changing environmental demands, making communities more accessible and accommodating for people with arthritis, which will foster their social participation [49].

### Building a community of support

The narratives from people living with arthritis were loud and clear on the need to build a community of peers that allows them to feel more supported and learn as a collective about how to live better with this chronic condition. Interestingly, building such a community was perceived as a more efficient strategy to create positive changes for people living with arthritis in Siksika than to keep an “individual” management approach. The need and effectiveness of peer support has been demonstrated in different populations living with chronic diseases as social relationships and support have “a long-standing association with health”, while social isolation has consistently predicted poor health outcomes [50].

The instrumental and emotional continuous supports provided by peers have been proved to be important to sustain behavior change and health for people living with various chronic diseases [50] and has proven beneficial for the control of chronic pain secondary to musculoskeletal diseases [51]. Our findings suggest that peer support provides validation of the “sick role” for individuals living with arthritis in this community, which allows them to accept their life situation. A qualitative meta synthesis on peer support interventions for chronic disease management, including arthritis, showed that one of the main effects of peer support is a sense of connection that allows for finding common meaning [52]. Therefore, finding common meaning could be the key process that explains our findings related with validating the sick role and seems to allow people to move forward accepting their chronic conditions and focus on living meaningful lives even when their health and function status are not optimal.

The need of building an arthritis community of support was not strongly identified in the narratives of providers/administrators, which could suggest a need for this group to better understand the social dimension of living with this chronic condition, especially the need for social validation. In fact, understanding the social aspects of living with arthritis has been identified as essential for improving the lives of people living with arthritis in vulnerable contexts [53]. Building a community of peers could allow for increasing social validation through a collective narrative repair process [54]. This narrative repair process consists on the creation of new narratives formed by strategic stories of health that promote actions that promote societal changes, involving the recognition and acceptance of arthritis as a genuine health problem that requires the support from all members of the community. Obtaining the collective power to conduct a narrative repair process, transforming the perceived sick role to a resilience champion role, will allow people with arthritis to effectively challenge existing beliefs about arthritis in Siksika and obtain desired community validation and support [54].

### Need for a community facilitator

Finding a need for a community facilitator as a solution to address the main needs identified during the participants’ narratives was not surprising, considering the nature of the in-depth interviews applied in this study, which purposefully explored the idea of a “case-manager model” as a potential solution to address patient’s necessities. Nevertheless, our results found differences in the perspectives about the potential role of a community facilitator between participants with arthritis and providers/administrators. For people with arthritis, a community facilitator was essential to increase illness understanding and get support to implement strategies to improve their health; while for providers/administrators the essential role of a community facilitator was related with aiding in the patient-provider communication and consistently monitoring disease-related outcomes. These perspectives are complementary and suggest that health literacy and good communication are essential prerequisites to care plan definition and implementation. In addition, implementation of care plans could be greatly facilitated by a constant and consistent illness monitoring that allows the identification of issues and solutions within existing health care systems.

The application of system navigation models for managing chronic diseases has shown effectiveness to improve models of health care [55]. In addition, there is evidence that patient support, case management and system navigation delivered by trained personnel who are trusted members of communities is cost-effective to manage some chronic diseases, especially in underserved, vulnerable communities [56]. Even though, system navigation strategies have not been amply explored in Canada, there is evidence of its effectiveness to improve health outcomes of immigrants living with chronic diseases in the United States [57]. In Alberta, it has been recognized that difficulties navigating the health system is one of the main barriers to improve the health of people living with diabetes [58].

Our findings suggest that a figure that can provide consistent care and system navigation support could be efficacious to improve health outcomes and service satisfaction. Furthermore, our interpretations suggest that the active ingredients for a successful implementation of such a model of care are disease education, self-management support and improved communication. It is also clear that the education component needs to incorporate Indigenous knowledge about health and healing along with knowledge on the local realities lived in Siksika and this education should be directed to both, people with arthritis and providers.

### Limitations

The use of a preconceived idea of how to improve the current arthritis model of care in Siksika, based on evidence on “case management”, influenced on the stories provided by participants. This influence potentially limited participants’ responses about relevant actions to improve care to those actions that had been reflected and accepted by the research team. Consequently, this somewhat limits the trustworthiness of our findings, suggesting there could be other solutions to improve the arthritis model of care in this community that were not considered. However, this limitation was softened by our member checking process, which allowed participants to provide further opinions on their care, after the accuracy of the interpretations was confirmed. In fact, the original researchers’ conceptualization of the case manager model changed and was shaped by participants’ perspectives. In addition, other needs outside the case management model were identified and interpreted in the results (i.e. group peer support, function based interventions), which suggests that the narratives elicited moved beyond the original proposal of a case manager model to improve the care of people living with arthritis in this reserve. The use of an authoritative voice in the formulation of results and interpretations has the risk of producing over interpretations that support researchers’ ideas and pre-conceptions. Consequently, by providing extensive quotations from participants’ narratives we intend to leave room for readers’ alternative interpretations of the data presented. It is important to mention that our findings are limited to the social reality of Siksika Nation, however there are similarities with other Indigenous reserves that could make these findings relevant and implementable in other Canadian Indigenous contexts.

### Recommendations to improve the current indigenous rheumatology model of care in Siksika Nation

1. To promote health providers’ reflections on the concepts of disability and how this is the result of not only disease processes, but the result of social structures that put Indigenous Peoples in situations of disadvantage.
2. To generate processes that allow increasing the cultural competence of health providers in collaboration with Siksika community.
3. To enact recommendation number 22 of the TRCC in Siksika by fostering and honoring a respectful integration of Siksika knowledge on the healing of people with arthritis with the support and guidance of Elders.
4. To increase the continuity of care for people with arthritis in Siksika through mechanisms that promote long-term and trust-based relationships between people with arthritis and their providers.
5. To establish strategies that can help increase community understanding of arthritis in Siksika and advocate for structural changes within the Nation to facilitate full community participation for people with arthritis.
6. To create a community of people living with arthritis in Siksika through a model that fosters communication, integration and collaboration.
7. To implement a community member-led process of care that incorporates the concepts of illness education, knowledge exchange, effective patient-provider communication, care plans, illness monitoring and system navigation, contributing to establish long-term trust-based relationships.

## Conclusions

Our study identified five main needs to improve the arthritis model of care in Siksika Nation: 1) implementing services that are more patient-centered and consider people’s functioning, 2) enhancing patient-provider communication, 3) improving continuity of care, 4) increasing community awareness on arthritis and 5) building arthritis peers support. These needs could potentially be addressed by the provision of services by a trained community member encompassing illness education, knowledge exchange, facilitation of patient-provider communication, support for care plans definition, illness monitoring and system navigation support, along with a community-led, culturally sensitive education strategy to increase health providers’ cultural competence. Enacting the seven specific recommendations that derived from this study in clinical practice has the potential to significantly improve the functioning, health and quality of life of people living with arthritis in this community. Central to these recommendations is the need to develop solid trust-based relationships where Indigenous and non-Indigenous knowledge converge to improve the individual and collective health of Siksika Nation.

## Supplementary Information

**Additional file 1.** Stakeholders Interview Guide.

**Additional file 2.** Patients Interview Guide.

**Additional file 3.** Stage One Analysis Logical Models.

## Data Availability

The qualitative dataset generated and analysed during the current study are not publicly available due to privacy policies, but de-identified data are available from the corresponding author on reasonable request.

## Abbreviations

ALS: Adalberto Loyola Sanchez
CB: Cheryl Barnabe
CIHR: Canadian Institutes of Health Research
CIORA: The Canadian Initiative for Outcomes in Rheumatology Care
DL: Diane Lacaille
ICF: International Classification of Functioning, Disability and Health
IPB: Ingris Pelaez-Ballestas
LC: Lindsay Crowshoe
OA: Osteoarthritis
Pos Doc: Post doctorate
RA: Rheumatoid arthritis
REB: University of Calgary Conjoint Health Research Ethics Board
RH: Rita Henderson
TW: Tyler Whyte
U: University
USAID: United States Agency for International Development
WHO: World Health Organization
