# Supplementary material for: “There are still a lot of things that I need”: a qualitative study exploring opportunities to improve the health services of First Nations People with arthritis seen at an on-reserve outreach rheumatology clinic
Source: BMC Health Serv Res. 2020 Nov 25;20:1076. doi: 10.1186/s12913-020-05909-9 (PMC7687986; doi:10.1186/s12913-020-05909-9)
Supplement: Supplementary file 1 — Additional file 1. Stakeholders Interview Guide. [file 12913_2020_5909_MOESM1_ESM.docx]

Stakeholders First Interview Round

Interview guide

***Objectives***

1. To identify stakeholders’ needs and desires for the case manager model.
2. To identify the role of a case manager according to different stakeholders.
3. To identify ideas from stakeholders about how to facilitate the case manager model sustainability considering existing health resources and funding institutions.
4. To identify stakeholders’ interests, expectations of the project and communication requirements.

***Introduction***

My name is Adalberto (Beto) Loyola Sánchez, I am a post doc at U of Calgary and I am working in a new project aimed at developing a case manager care model to improve the health care of people living with arthritis and other chronic diseases in the Siksika Nation Reserve.

At this moment, I am collecting stories and points of view from important people (stakeholders) like you about how a case manager model of care for people living with arthritis and other chronic diseases could and should work. Also, I am collecting perspectives on how can we make this case manager model sustainable over time.

Do you agree to have this conversation with me? (Obtain verbal consent) if yes ask if it is okay with you if I audio record our conversation? (If yes record consent).

***Interview content***

1. Personal story *(the purpose is to have context of whom is providing us information and also have an idea of the interests of all stakeholders interviewe*d), Let’s talk a little about yourself, who you are and why are you doing your current job? **Prompts: interests, disinterests, personal drivers and motivators, specifically interests on First Nations Health**.
2. Please consider the following statements: *1) First Nations peoples of Canada lack optimal access to many health services, especially people living with arthritis and other chronic diseases; and 2) Case manager models may improve access to appropriate healthcare through selecting a person from the community, who provides patient support services and develops community supports*.
3. Reflection based on the two previous statements.
   1. Considering your personal experience, what are your first thoughts and reactions to these statements? **Prompts: could a CM be beneficial for you? Could this be beneficial for people living in Siksika? And why?**
   2. Case Manager Responsibilities and Skills
      1. Who would be the ideal type of person to execute the role of a case manager? **Prompts: Personal characteristics and skills**.
      2. What should a case manager do? **Prompts: in relation to a) patients and family members, b) existing services and service providers, and c) traditional medicine. What does a case manager could do to facilitate your work? Amount of time necessary to perform a case manager role.**
   3. Case manager model sustainability
      1. For participants in an administrative/oversight/leading role:
         1. What are the existing financial arrangements to provide health services in Siksika (or Indigenous communities in Alberta)?
         2. How can a case manager model be sustainable over time? **Prompts: financial arrangements, case manager salary**.
         3. What are the most important factors that your organization takes into consideration for making decisions? **Prompts: financial decisions, budget spending; what would your organization need to know in order to consider financing a case manager model?**
      2. For participants in a service providing role:
         1. What are the existing financial arrangements that support your position at the Siksika Health and Wellness Centre? **Prompts: salary, job accountability.**
         2. Considering your experience, how can a case manager position be sustainable over time? **Prompts: what would the case manager model have to do for you to support it and use it in the long run? Case manager salary.**
      3. For participants in a user role:
         1. What would the case manager service have done for you in order for you to use it and support it in the long run? **Prompts: expectations on a) service characteristics, b) attended needs, c) time spent per week providing services.**
4. **For Blackfoot people**: Can you explain me the traditional ways for making community decisions? **Prompt: The meaning of OKAHTSIIMAHN (described in the agreement with AHS). For No-Blackfoot people:** Would you support the creation of a case manager model for people living with arthritis + chronic diseases in Siksika (Indigenous communities)? Why?
5. What would be your expectations from this project? **Prompts: would you like to be informed about what is happening in this project? What is the best way to communicate with you? What are the outcomes that you expect from this project**? **Are you willing to participate in a group meeting to get informed about the results of these interviews and make some decisions on the project?**
6. Are there other people I should talk to about this? Any other thoughts about the case manager model?
7. Thank you. End of interview.
