# Supplementary material for: “There are still a lot of things that I need”: a qualitative study exploring opportunities to improve the health services of First Nations People with arthritis seen at an on-reserve outreach rheumatology clinic
Source: BMC Health Serv Res. 2020 Nov 25;20:1076. doi: 10.1186/s12913-020-05909-9 (PMC7687986; doi:10.1186/s12913-020-05909-9)
Supplement: Supplementary file 2 — Additional file 2. Patients Interview Guide. [file 12913_2020_5909_MOESM2_ESM.docx]

Patients First Interview Round

Interview guide

***Objectives***

1. To identify patients’ needs that can be addressed through a case manager/system navigator model.
2. To help define the case manager/system navigator role.
3. To identify the health system gaps from the perspective of the users.
4. To identify and recruit patients to engage in the decision-making processes of the project.

***Introduction***

My name is Adalberto (Beto) Loyola Sánchez, I am a post doc at U of Calgary and I am working in a new project aimed at developing a model of care to improve the health of people living with arthritis and other chronic diseases in Siksika.

At this moment, I am collecting stories and points of view from people living with arthritis like you about how the ***model should work***.

Do you agree to have this conversation with me? If yes, hand in the consent form and offer help to go through it. After reading this, do you agree to participate? If yes, sign consent form and remind that the conversation will be audiotaped.

***Interview content***

1. Personal story *(The purpose is to obtain the narrative of the illness including its trajectory as a way to prepare the ground to reflect on needs*), Please share with me your story. Who you are? and How is it like to live with your arthritis and other conditions (if applicable) ? **Prompts: what diseases do they have, since when, how they presented, how do they shape their live-experience**, **ILLNESS TRAJECTORY DESCRIPTION specifically addressing the no-show ups and the difficulties in communication with the Rheumatologists clinic.**
2. Considering your story, what are your main needs right now? **Prompts: what is missing, what would you like happening with your life and with your care**.
3. Considering your story, what are your main strengths? How can you use these strengths to address these needs?
4. We are thinking of hiring someone to help people like you to obtain their needs and improve their health. We know that in other places and with other diseases it has been beneficial to have a person giving support to patients. This support has included:
   1. Providing information, emotional support, and education to health providers about a specific culture.
   2. Acting as a mediator between the people and the health providers in order to solve differences and misunderstandings.
   3. Advocating to cover people’s needs through:
      1. Linkage to required services
      2. Practical assistance
      3. Facilitating decision making
   4. Building trust between patients and providers.
5. What do you think about this? **Prompts: does this make sense to you, do any of these actions could be important for you, and would you like to have something like this.**
6. If we hire a person to do these types of things, what is more important to be done why and how should it be done? **Prompts: if the narrative is scarce go action by action (a to c in point 4) and ask specifically about importance and the way it could be done.**
7. **Would you be willing to participate in a group to make decisions on how the model will work and how are we going to evaluate it?**
8. Do you think there are other people living with arthritis I should talk to about this?
9. Any final thoughts about what we discussed?
10. If after analyzing what you said I have some questions or want to clarify something, can I contact you again? Thank you. End of interview.
