# Supplementary material for: “There are still a lot of things that I need”: a qualitative study exploring opportunities to improve the health services of First Nations People with arthritis seen at an on-reserve outreach rheumatology clinic
Source: BMC Health Serv Res. 2020 Nov 25;20:1076. doi: 10.1186/s12913-020-05909-9 (PMC7687986; doi:10.1186/s12913-020-05909-9)
Supplement: Supplementary file 3 — Additional file 3. Stage One Analysis Logical Models. [file 12913_2020_5909_MOESM3_ESM.pptx]

## Slide 1
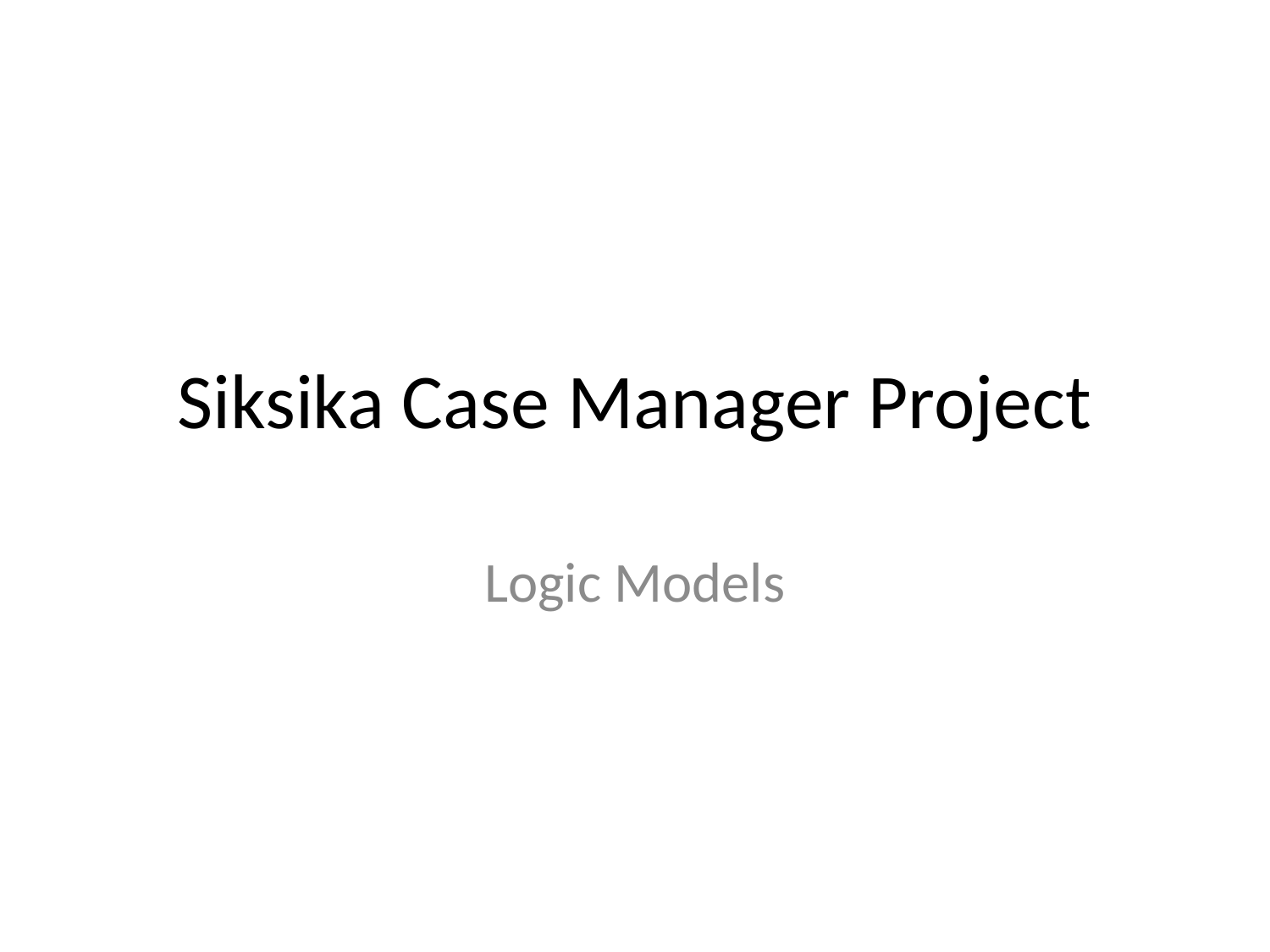

# Siksika Case Manager Project
Logic Models

## Slide 2
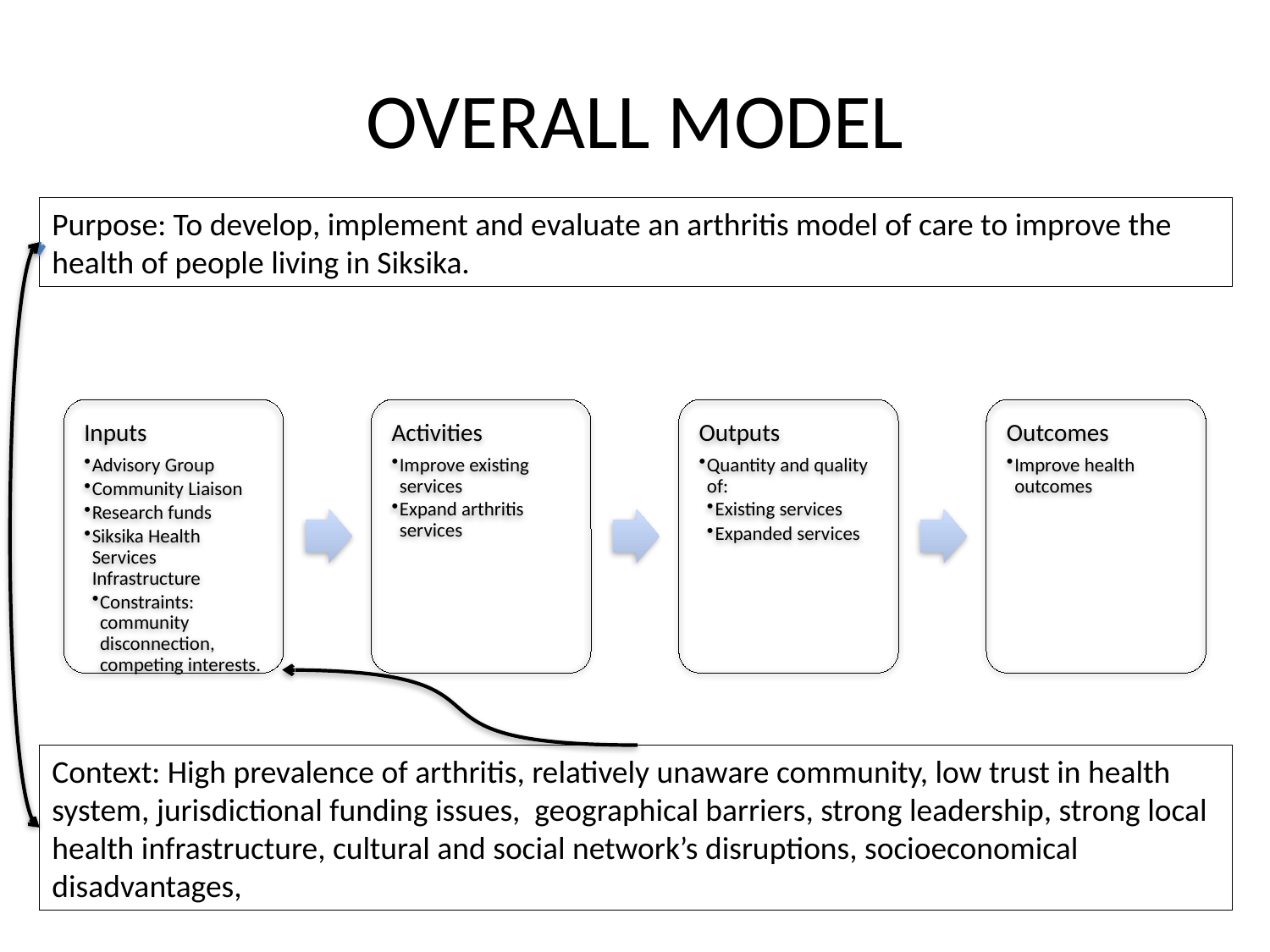

# OVERALL MODEL
Purpose: To develop, implement and evaluate an arthritis model of care to improve the health of people living in Siksika.
Context: High prevalence of arthritis, relatively unaware community, low trust in health system, jurisdictional funding issues, geographical barriers, strong leadership, strong local health infrastructure, cultural and social network’s disruptions, socioeconomical disadvantages,

## Slide 3
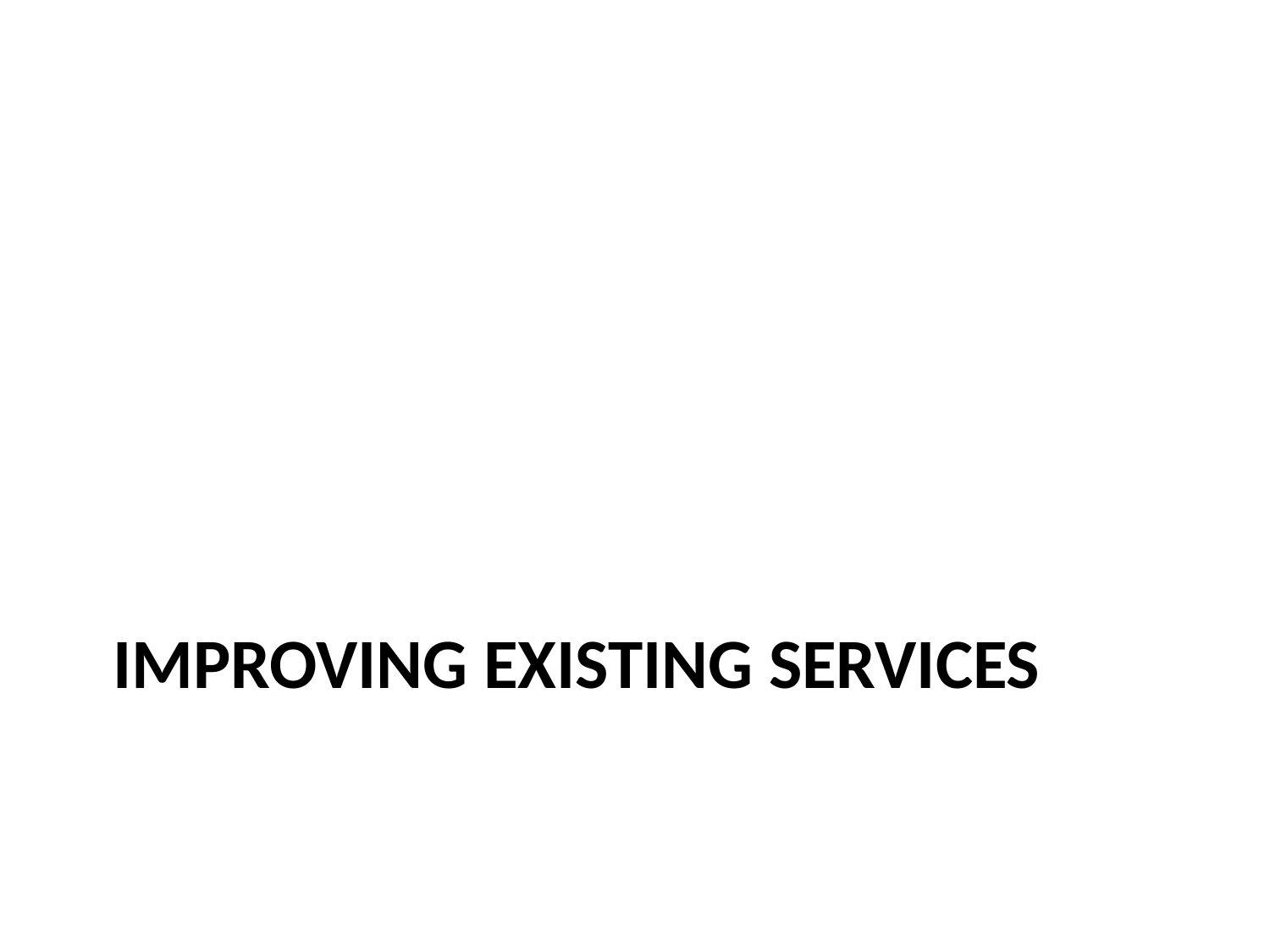

# Improving existing services

## Slide 4
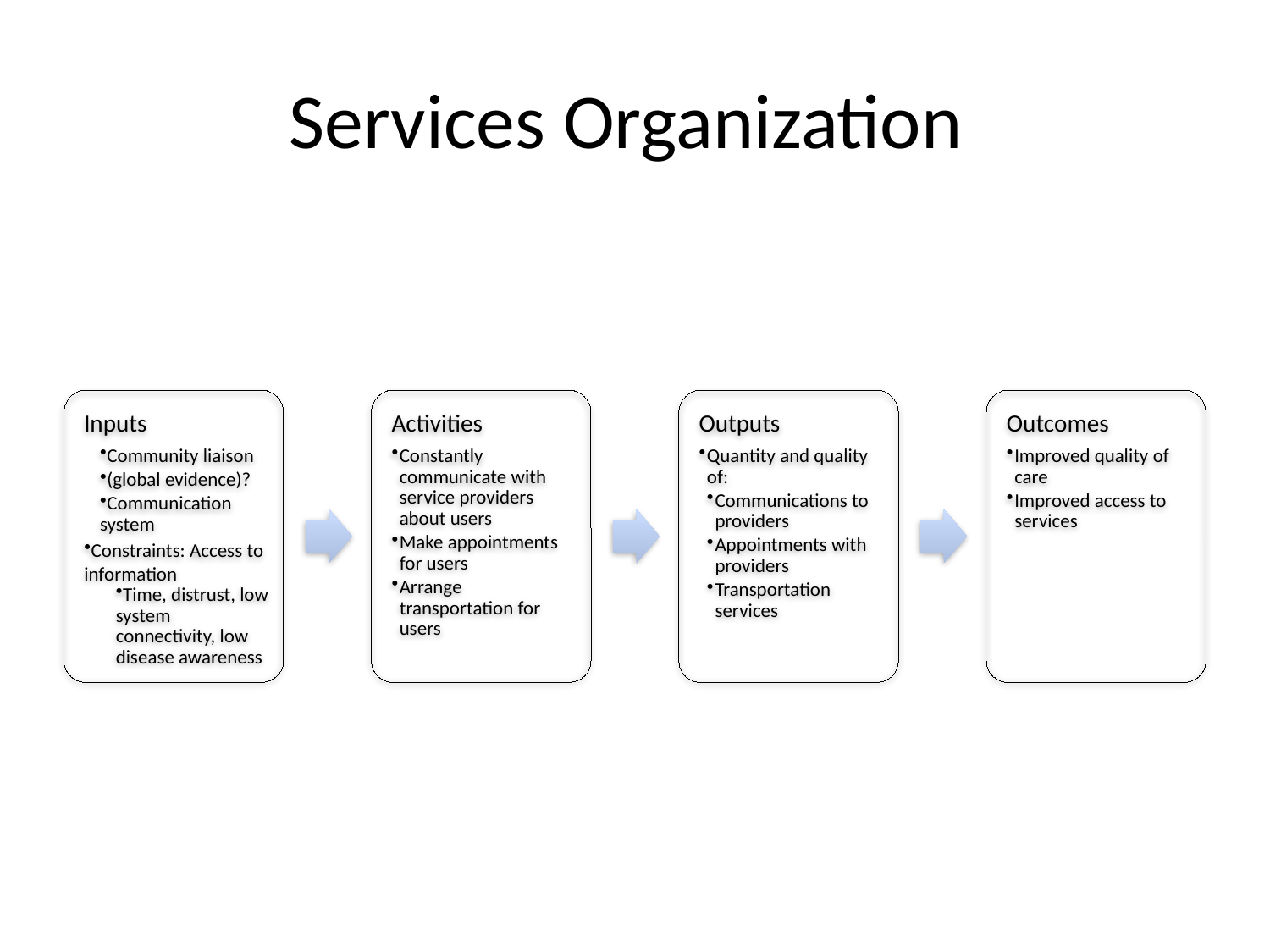

# Services Organization

## Slide 5
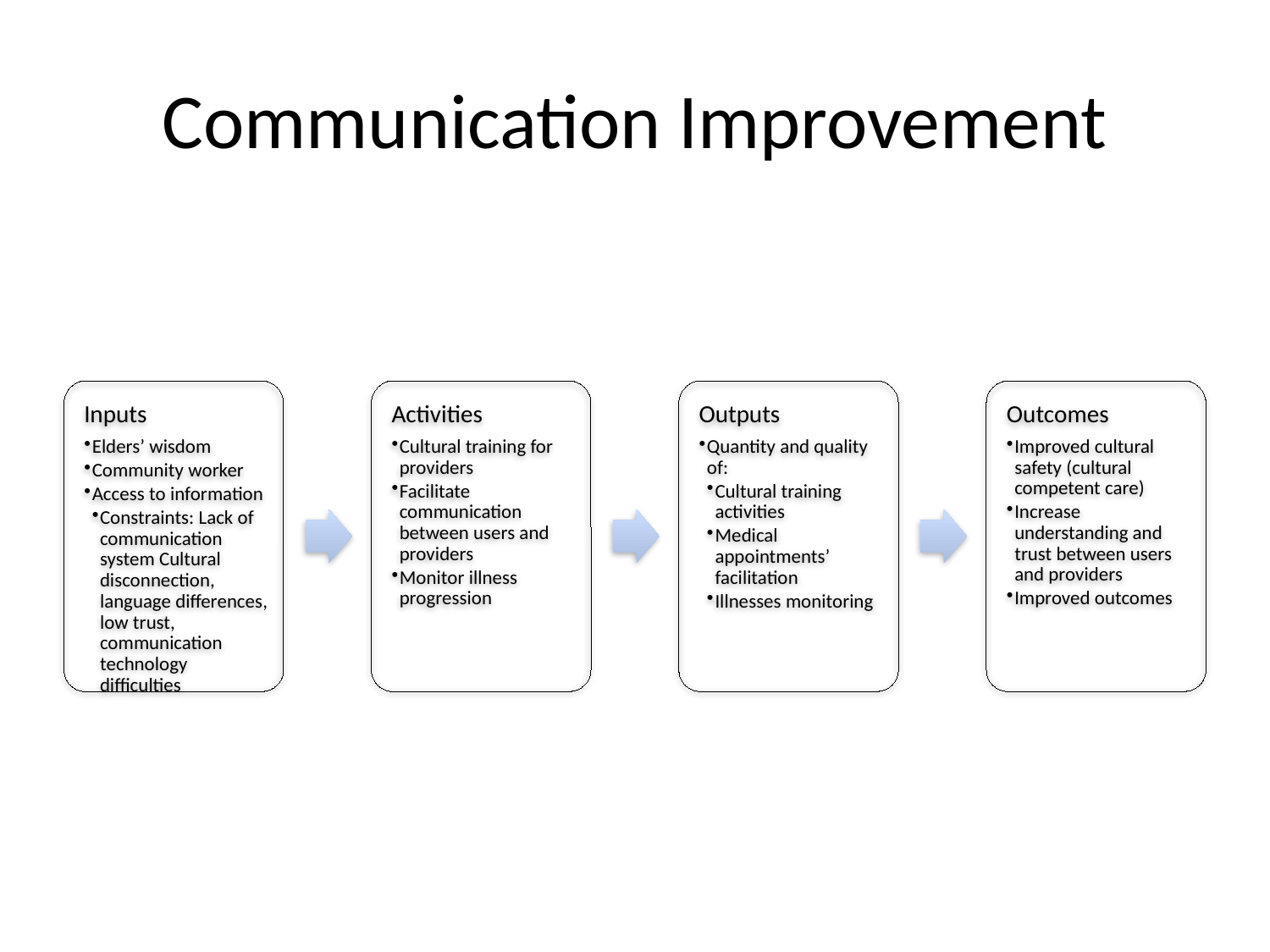

# Communication Improvement

## Slide 6
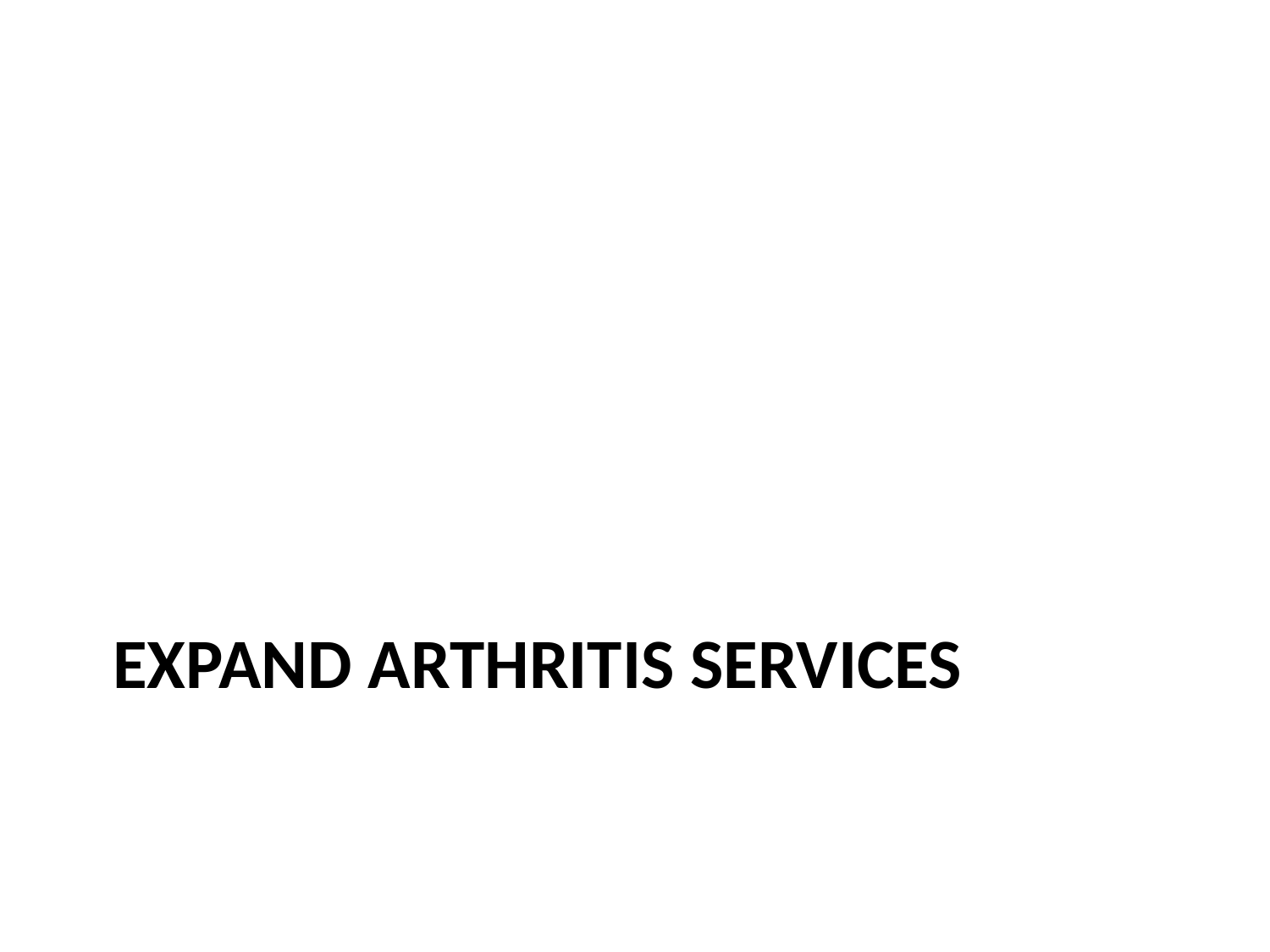

# EXPAND ARTHRITIS SERVICES

## Slide 7
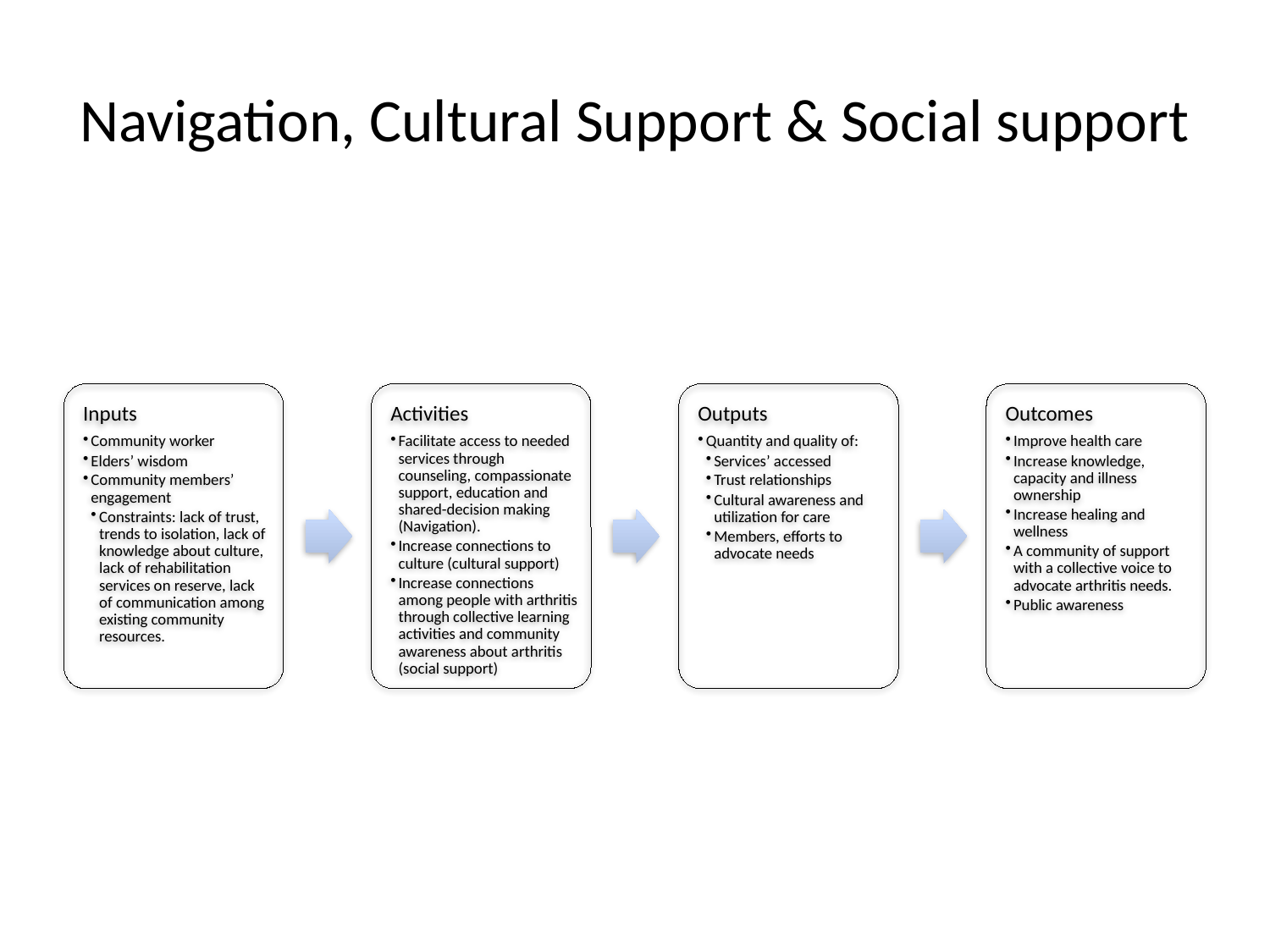

# Navigation, Cultural Support & Social support
